# Social innovation, goal orientation, and openness: insights from social enterprise hybrids

**DOI:** 10.1007/s11187-022-00643-4

**Published:** 2022-07-01

**Authors:** John Hagedoorn, Helen Haugh, Paul Robson, Kate Sugar

**Affiliations:** 1 School of Business and Management, Royal Holloway University of London, Egham, TW20 0EX UK; 2 UNU-MERIT, Maastricht University, Maastricht, the Netherlands; 3 Judge Business School, University of Cambridge, Trumpington Street, Cambridge, CB2 1AG UK; 4 Bath, UK

**Keywords:** Social innovation performance, Openness, Social enterprise hybrids, Social enterprise goals, L31, O35, O36, L39, D29, C12, C33

## Abstract

We empirically examine social innovation and openness through a survey of social enterprise hybrids in the United Kingdom (UK). Social innovation refers to new products, processes, and services that respond to grand challenges. Social enterprises pursue economic, social, and environmental goals but vary in their goal orientation, namely the relative importance ascribed to such goals. We first explore the relationships between commercial, social, and environmental goal orientation and social innovation performance. Next, we consider the moderating impact of openness to external knowledge and ideas on social innovation performance. Our analysis finds positive and significant relationships between commercial and social goal orientation and social innovation performance, but no relationship with environmental goal orientation. In addition, the use of external sources of knowledge and ideas positively strengthens these relationships for both commercial and social goal orientation but not for environmental goal orientation. Our results reveal some important influences on social innovation, openness, and hybrid organizing.

## Abstract (plain language summary)

Headline: The more social enterprises focus on both commercial and social goals, the more successful they are in improving their social innovation performance.

Social innovation refers to new products, processes, and services that respond to a range of social challenges such as poverty, inequality, homelessness, health, and environmental issues.

Our study suggests that the more social enterprises focus on both commercial and social goals, the higher their social innovation performance. In addition, the more open innovation-oriented social enterprises are, that is, the more they use external sources of knowledge and ideas, the more they can benefit from their commercial and social goals to improve their social innovation performance.

Implications of our research for practice: social enterprises are encouraged not only to focus on both commercial and social goals but also to build relationships with external stakeholders. These external stakeholders can provide information on entrepreneurial opportunities, how to respond to problems and market and government failures, and how to remain successful while collaborating with a range of partners.

## Introduction

Social innovation has emerged as a new variant of innovation in which the aim is to generate new products, processes, or services to address the grand challenges of poverty and inequality and community issues such as homelessness and health deficiencies, as well as environmental issues related to climate change and pollution, and the use of recycling and reusing, and green energy production (Adams & Hess, 2010; Bogers et al., 2017; Choi & Majumdar, 2014; Eichler & Schwartz, 2019; Kickul et al., 2013; Moulaert et al., 2013; Mulgan, 2006; Phills et al., 2008; Tracey & Stott, 2017; van der Have & Rubulcaba, 2016; van Wijk et al., 2018). Despite some definitional ambiguity (Adams & Hess, 2010; Edwards-Schachter & Wallace, 2017; Eichler & Schwartz, 2019; Lawrence et al., 2015), the potential of social innovation to resolve social and environmental problems has been widely celebrated (Baskaran & Mehta, 2016; Molecke & Pinkse, 2017; Parrish, 2010; Phills et al., 2008; The Young Foundation 2012). Prior analyses of social innovation have been dominated by case studies (e.g. Bhatt et al., 2016; Datta, 2011; Eichler & Schwartz, 2019) and qualitative field studies that analyse data gathered via direct observation and interviews (Giudici et al., 2020), complemented by insights from practitioners and policy makers (The Young Foundation 2012). Reviews of social innovation have brought together this diversity of literature and identified important areas where empirical research would be most valuable (Eichler & Schwartz, 2019; Foroudi et al., 2021; Lawrence et al., 2015; Phillips et al., 2015; Tracey & Stott, 2017; van der Have & Rubulcaba, 2016). In this paper, we build on these recommendations and focus on the links between social innovation performance, social enterprise goal orientation, and openness to external sources of knowledge and ideas.

The concept of openness describes how external knowledge and ideas are employed by firms to improve their performance (McCartt & Rohrbaugh, 1995) and promote innovation (Chesbrough & Bogers, 2014; Love et al., 2014). Previous empirical investigations have operationalized openness in a variety of ways, however, most scholars agree that openness is a measure of the extent of an organization’s use of external sources of information to enable innovation (Chesbrough et al., 2006; Dahlander & Gann, 2010; Lazzarotti et al., 2011). Openness is defined as the breadth and depth of external sources of information for innovation in which breadth refers to the diversity and depth the importance of sources (Leiponen & Helfat, 2010; Zobel et al., 2017). In our paper, we conceptualize openness as an inbound measure of the extent to which the use of external knowledge and ideas is related to impacts on social innovation performance. Our conceptualization of openness thus aligns with how organizations use “a wide range of external actors and sources to help them achieve and sustain innovation” (Laursen & Salter, 2006, p. 131).

Research has investigated the influence of openness in private sector firms (e.g. Laursen & Salter, 2006) and non-profit sector organizations (e.g. Holmes & Smart, 2009). While prior research suggests that relationships with stakeholders influence social innovation (Phillips et al., 2015, 2019), we have yet to fully understand how openness influences innovation in hybrid organizations that blend characteristics of both commercial and non-profit organizations (Battilana & Lee, 2014; Doherty et al., 2014). This context is intriguing because the firm-level blending of logics from different economic sectors suggests that social enterprise hybrids may be uniquely positioned to capitalize on openness to knowledge and ideas from external sources in different domains.

Prior research has empirically examined the relationship between openness and innovation in manufacturing firms (e.g. Amara & Landry, 2005; Drechsler & Natter, 2012; Laursen & Salter, 2006) and service organizations (e.g. Hidalgo & D’Alvano, 2014; Mina et al., 2014; Rubulcaba et al., 2012). The results endorse the importance of external sources of knowledge and ideas, and networks in particular (Nieto & Santamaría, 2007; Zeng et al., 2010). Studies of organizations in the non-profit sector have examined innovation in universities (Perkmann & Walsh, 2007) and charities (Holmes & Smart, 2009; McDonald, 2007) and further endorse the importance of inbound knowledge and ideas on innovation. By responding to calls for more research to better understand innovation in non-profit (Chesbrough & Minin, 2014; West et al., 2014) and hybrid organizations (Battilana & Lee, 2014; Eichler & Schwartz, 2019; Wilson & Post, 2013), and social innovation implementation (Phillips et al., 2019), our research advances knowledge on external influences on social innovation performance.

In our study, we analyse data gathered from community interest companies (CICs)—a novel type of social enterprise hybrid introduced in the United Kingdom (UK) in 2005 (Haugh, 2021; Haugh et al., 2022)*.* Social enterprise hybrids are organizations where the social logic of non-profit organizations is blended with the commercial logic of for-profit enterprises (Battilana & Lee, 2014; Doherty et al., 2014; Martin & Osberg, 2007; Stevens et al., 2015). In other words, social enterprise hybrids are a distinct category of organizations because they combine the features of both for-profit enterprises and non-profit organizations. In contrast to the private sector mission to create and capture personal or shareholder wealth (Phillips et al., 2015; Phills et al., 2008; Santos, 2012), the mission of social enterprise hybrids is to achieve financial sustainability, social and environmental impact, and advance social change (Battilana & Lee, 2014; Bhatt et al., 2016; Doherty et al., 2014; Mair & Martì, 2006; Stevens et al., 2015). Social enterprise hybrids, however, vary in goal orientation, namely the relative importance ascribed to commercial, social, and environmental goals (Mair & Martì, 2006).

There are approximately 70,000 social enterprise hybrids in the UK (Villeneuve-Smith & Temple, 2015) and the continuing increase in their numbers (Amin et al., 2002; Defourny & Nyssens, 2010) has attracted the attention of scholars, practitioners, and policy makers keen to explain how such organizations concurrently pursue multiple goals (Cukier et al., 2011; Dacin et al., 2011; Lepoutre et al., 2013; Phillips et al., 2019; Stevens et al., 2015). Explanations for this increase in the population of social enterprise hybrids comprise both supply and demand factors. Financial austerity has created opportunities for the establishment of new social enterprise hybrids to bid for contracts to deliver out-sourced public services (Brandsen et al., 2005; Chell, 2007; Doherty et al., 2014; Evers, 2005; Fawcett & Hanlon, 2009; Graddy-Reed & Feldman, 2015; Haugh & Kitson, 2007; Perrini et al., 2010; Uyarra et al., 2014; Vickers et al., 2017; Voorberg et al., 2015). At the same time, entrepreneurs have responded to deficiencies in economic justice and rising societal inequality by turning to the establishment of social enterprise hybrids to address market and government policy failures and transform society (Alvord et al., 2004; Austin et al., 2006; Di Domenico et al., 2010; Doherty et al., 2014; Van Sandt et al., 2009; Van Wijk et al., 2018). Much of the social entrepreneurship literature has praised the innovativeness of social enterprise hybrids (Chalmers & Balan-Vnuk, 2012; Zahra et al., 2009) but there has been little theorization and theory-driven empirical research to substantiate this claim (Liu et al., 2015; Phillips et al., 2019).

Situating our research in the social innovation and openness literatures, we first develop hypotheses on the relationships between commercial, social, and environmental goal orientation and social innovation performance, in terms of the extent to which social innovation activities affect societal transformation. Next, we hypothesize the moderating effect of openness to external sources of knowledge and ideas on these relationships. Our hypotheses are tested on data gathered from a proprietary survey of social enterprise hybrids. Our results indicate that both commercial and social goal orientation are positively related to social innovation performance and that openness to external sources of knowledge and ideas strengthens these relationships. The relationship between environmental goal orientation and social innovation performance, however, is negative and not strengthened by openness to external sources of knowledge and ideas.

The paper makes three contributions to the literature. First, in the context of the social innovation literature, our findings suggest that commercial and social goal orientation are positively and significantly related to social innovation performance. Thus, social enterprise hybrids that have developed strategies to become commercially successful and, as such, to achieve financial sustainability as well as to create social impact are also successful social innovators. Social enterprise hybrids that pursue a broader set of environmental goals, however, are not successful social innovators and are, due to their technologically more complex nature, more likely to be social innovation imitators.

Second, our research contributes to the openness literature as we find that social enterprise hybrids’ use of external sources of knowledge and ideas moderates social innovation performance. Prior descriptions have emphasized the participation of users in social innovation idea generation and implementation (Phillips et al., 2019; Rodin, 2010; Voorberg et al., 2015) since beneficiaries are argued to have first-hand knowledge of a social problem (Lawrence et al., 2015; Svensson & Bengtsson, 2010), and accrued greater legitimacy (Dart, 2004), and this helps to achieve customer loyalty, satisfaction, and, in turn, competitive advantage (Voorberg et al., 2015). For example, citizens are involved in co-creation, re-design, and transformation of health care and education services in the public sector (Brandsen & Pestoff, 2006; Voorberg et al., 2015), and the homeless are involved in contributing to content and selling the Big Issue newspaper (Hibbert et al., 2002). Our analysis finds that openness to external sources of knowledge and ideas strengthens the relationship between commercial and social goal orientation and social innovation performance.

Third, taken together, the results also shed new light on the current conception of hybrid organizing which to date has predominantly focussed on internal management processes (Battilana & Lee, 2014) and neglected the influence of external relationships on organizational processes, specifically social innovation (Phillips et al., 2019). Openness to external sources of information and knowledge broadens the horizon of organizations and increases the likelihood of greater social innovation by social enterprise hybrids.

## Theory development and hypotheses

### Social innovation

Social innovation describes the adoption of “new solutions to social problems” (Tracey & Stott, 2017, p. 51) or to environmental problems (Dawson & Daniel, 2010) that impact on society (Grieco et al., 2015) and social innovation practice has happened ahead of research and theory development (Mulgan, 2015). The two key constructs are the development of a new solution and that the benefits are shared beyond the innovating organization to impact, or transform, society (Bhatt et al., 2016; Foroudi et al., 2021; Tracey & Stott, 2017; van Wijk et al., 2018). Social innovation, for example, includes new products, processes, and services that respond to grand challenges of poverty and inequality, as well as community issues such as homelessness and health deficiencies (Christensen et al., 2009; Graddy-Reed & Feldman, 2015; Jensen & Fersch, 2019; Mair et al., 2012; van der Have & Rubulcaba, 2016). Social innovation also includes finding new solutions to environmental problems, such as the development of new products, processes, and services to address climate change and pollution, fostering eco-behaviour such as recycling and reusing and green energy production (Berrone et al., 2013; Brunnermeier & Cohen, 2003; Ongondo et al., 2013; Vickers & Lyon, 2014; Voorberg et al., 2015). Social innovation is “good for society and enhances society’s capacity to act” (Eichler & Schwartz, 2019, p. 533; see also Murray et al., 2010) and thus offers a new perspective to mainstream innovation studies by bringing social and environmental impact into consideration (Alvord et al., 2004; Bhatt et al., 2016; Foroudi et al., 2021; Voorberg et al., 2015). Furthermore, innovating to address market and government policy failures (Lettice & Parekh, 2010; Mulgan, 2006; Phillips et al., 2019) and creating social transformation (Cajaiba-Santana, 2014) substantially distinguish social innovation from conventional business innovation (Foroudi et al., 2021). For example, Huq’s (2019) review of the social innovation literature found that the majority of the research takes place in either a setting of political, economic, and/or social turmoil; or, where market institutions are weak or absent.^1^ For example, the Jensen and Fersch (2019) study of novel forms of elder care in Denmark, and Tracey et al. (2011) examined how employment creation helped the homeless to earn income.

The demand for social innovation may be derived from market failures when organizations do not satisfy demand for specific products and services (Domenico et al., 2010Santos, 2012), such as customer demand for products that create positive social and environmental impacts, e.g. fair trade (Doherty et al., 2013). Demand for social innovation may also be derived from opportunities created when the public sector does not satisfy demand for social and welfare services (Santos, 2012), e.g. social housing (Blessing, 2012). By implementing public procurement of social and welfare services, public policy provides a mandate to encourage social innovation (Volkmann et al., 2021). Moreover, employees may be attracted to employers with pro-social and environmental credentials (Radoynovska & Ruttan, 2022).

The supply of social innovation may also be influenced by several factors, such as when organizations respond to societal expectations that prioritize sustainability and expect their products and services to create social value (Haigh et al., 2015) and not harm the environment (Bansal & Roth, 2000), for example to provide socially innovative access routes into meaningful employment in work integration (Battilana et al., 2015) and financial inclusion through access to microfinance (Battilana & Dorado, 2010). In addition, personal factors, such as life experience (Corner & Ho, 2010), emotions (Katre & Salipante, 2012), pro-social motivation (Bacq & Alt, 2018; Munoz et al., 2020), and compassion (Miller et al., 2012) encourage the establishment of prosocial organizational forms (Bastida et al., 2022) and the supply of social innovation.

Although not the sole preserve of social enterprise hybrids (Dietrich et al., 2016; Eichler & Schwartz, 2019; Tracey & Stott, 2017), social innovations are developed and implemented by organizations motivated by social mission (Mulgan, 2006). The range of organizational forms that pursue social innovation includes corporations (Herrera, 2015; Mirvis et al., 2016), public institutions (Carrie & Seddon, 2014; Rana et al., 2014), nongovernmental organizations (NGOs), and civil society organizations (Franklin et al., 2017). Our study investigates the Community Interest Company, an organizational form for locally-embedded social enterprises in the UK.

Many claims have been asserted concerning the innovativeness of social enterprise hybrids (Chell et al., 2010; Phills et al., 2008) much of which is derived from case studies of social innovations (e.g. Hibbert et al., 2002; Neumeier, 2012; Phillips et al., 2019; Phills et al., 2008; Seyfang, 2004; Have and Rubulcaba, 2016; Vickers et al., 2017) and descriptions of high profile social innovators (Dacin et al., 2011), such as Muhammad Yunus, the inventor of microfinance (Cajaiba-Santana, 2014), and John Bird, the founder of the Big Issue street newspaper (Hibbert et al., 2002). In contrast to a profit maximization goal, social innovation describes the impact of novel products, services, and processes that respond to social needs that would otherwise not be met (Phills et al., 2008) and that create social value beyond the capability of existing systems (Adams & Hess, 2010; Cajaiba-Santana, 2014; Phills et al., 2008; Westley & Antadze, 2010). Thus, it is the additional community and societal impacts and societal transformation potential of social innovation performance which is driving opportunity recognition and exploitation (Adams & Hess, 2010; Pol & Ville, 2009) and not commercial success per se (Phillips et al., 2019). Establishing the assumed innovation performance of social enterprise hybrids makes a useful contribution to theory and practice because in the increasingly competitive market for organizational funding, successful social innovators shed light on how to best secure and allocate scarce resources. We argue that social innovation performance is at the vanguard of the measures available to capture social and environmental impact and could be associated with greater efficiency, value creation, and societal transformation.

### Openness

Openness describes the organizational shift from investing internally, such as in research and development, to sourcing external knowledge and ideas to sustain innovation (Chesbrough, 2003; Fey & Birkinshaw, 2005; Laursen & Salter, 2006; Rodriguez et al., 2017). The openness paradigm has gained traction as the importance of external networks to firm-level innovation has been recognized (Freel, 2005; Laursen & Salter, 2006; Love & Roper, 2001; Nieto & Santamaría, 2007; Rothwell et al., 1974; von Hippel, 1988; Zeng et al., 2010). The acquisition of external knowledge and ideas from interactions with stakeholders such as relatives, suppliers, customers, and other organizations are key variables in the design of strategic innovation policies (Chesbrough, 2003; 2006a, 2006b). External knowledge and ideas are noted to be critical for private sector firm innovation (Cohen & Levinthal, 1990) in different national contexts, such as Canada (Amara & Landry, 2005), China (Zeng et al., 2010), Finland (Leiponen, 2000, 2005), Spain (Nieto & Santamaría, 2007), and Taiwan (Chiang & Hung, 2010). While there is an expanding literature that links together openness, innovation, and SMEs (Drechsler & Natter, 2012; Laursen & Salter, 2006), our knowledge of such connections in a social enterprise context is limited (Phillips et al., 2019). The focus of prior research has primarily been on private sector organizations, but how does the relationship between openness and innovation play out in social enterprise hybrids where the mission is to pursue commercial, social, and environmental goals?

For firms steeped in a paradigm of closed innovation, the transition to adopting an openness perspective on innovation is undoubtedly challenging (Alexy et al., 2013; Henkel et al., 2014). Yet, openness to external knowledge and ideas has many advantages over internally-focused innovating (West & Bogers, 2014, 2017). For social enterprises with an ethos of either a non-profit maximizing approach or a down-played focus upon profits compared to other goals, openness may not necessarily be as disruptive as for commercial firms (Dahlander & Gann, 2010).

Also, for social innovation, important questions to ask concern where ideas originate from and why some ideas flourish while others fall by the wayside (Mulgan, 2015). Van der Have and Rubulcaba (2016) in their review of 172 publications conclude that social innovation is grounded in a broad range of community and social settings. Relatedly, and in line with the current openness literature, prior commentaries of social innovation noted openness to knowledge flows from networks and close engagement with external stakeholders as important causal factors of social innovation success (Adams & Hess, 2010; Chesbrough & Minin, 2014; Mulgan, 2006).

### Hypotheses

Social enterprise hybrids’ mission is to create social value simultaneously with being commercially oriented (Choi & Majumdar, 2014; Santos, 2012). The context of social enterprise hybrids is generally described as resource-constrained (Chalmers & Balan-Vnuk, 2012) and characterized by market and government policy failures (Doherty et al., 2014). Such conditions foster the creative search for new solutions to social and environmental problems. To be commercially sustainable, social enterprises need to stay competitive and invest in continuous innovative capability development (Weerawardena & Mort, 2006). Moreover, social enterprise funding sources comprise a blend of unrestricted income, e.g. earned income and donations, and restricted income, e.g. grants and public sector contracts (Doherty et al., 2014). The generation of income from trading provides a flow of unrestricted funds into the organization, the surplus of which can be invested in social innovation.

By also focusing on commercial goals, social enterprise hybrids enact the market logic of entrepreneurial organizations—they innovate goods and services that meet the needs of customers. This enables social enterprise hybrids to both generate trading income and achieve their mission to serve communities. Thus, we would expect that the stronger the commitment to generating commercial income, the more financial resources are available for investing in social innovation and improving social innovation performance.

H1. In social enterprise hybrids, commercial goal orientation is positively related to social innovation performance.

Previous work indicates that social enterprise hybrids are established to serve markets where the private sector either cannot make a profit or lacks sufficient knowledge to design products, processes, and services to meet user needs (Austin et al., 2006; Moizer & Tracey, 2010). Similarly, the failure of public sector organizations to meet the needs of customers and beneficiaries has been attributed to government failure (Kerlin, 2006; Moizer & Tracey, 2010; Teasdale, 2012). To respond to failures in product, process, and service provision, social enterprise hybrids work closely with stakeholders to understand the types of products and services demanded by the market and to determine the best way of directing limited resources to and designing products, processes, and services that are sensitive to the operating context and responsive to the needs of beneficiaries (Chesbrough & Minin, 2014; Mulgan, 2006; Pache & Santos, 2012). Commitment to social mission may also be employed to leverage the flow of commercial income from trading, as in the case of fair trade certified producers and manufacturers (Doherty et al., 2014).

Social enterprise hybrids are expected to engage in developing innovative and creative solutions to social needs (Weerawardena & Mort, 2006) and the legitimacy of social enterprise hybrids is tied to their societal impact (Dart, 2004; Luke & Chu, 2013). Thus, we would expect that social enterprise hybrids that understand how social needs can be effectively and creatively responded to will design new ways to respond to social needs and increase social innovation performance.

H2: In social enterprise hybrids, social goal orientation is positively related to social innovation performance.

In addition to commercial and social goal orientation, social enterprise hybrids also respond to environmental market failure (Mair & Martì, 2006) and commit to environmental goal orientation (Doherty et al., 2014; Seyfang et al., 2014). For some social enterprise hybrids, the new products, processes, and services sold to commercial markets are directly related to the environmental mission, e.g. ICT reuse (Ongondo et al., 2013), green energy production (Huybrechts & Haugh, 2017; Seyfang et al., 2014) and eco-living communities (Kunze, 2012). In other organizations, the environmental mission is pursued indirectly, e.g. supporting environmental initiatives in the workplace and communities (Thompson & Doherty, 2006).

Governments have played an enabling role in shaping the regulatory environment and encouraging investment in the technologies for responding to environmental market failures (Vickers & Lyon, 2014). Financial incentives for renewable energy, waste management, and low-carbon technologies have stimulated interest in and adoption of green technologies. For example, government subsidies have supported the establishment of renewable energy cooperatives in Europe (Huybrechts & Haugh, 2017) and adoption of renewable energy sources in off-grid rural communities (Sengupta et al., 2020). Given these developments, we expect that the extent of environmental goal orientation of social enterprise hybrids is related to their social innovation performance.

H3: In social enterprise hybrids, environmental goal orientation is positively related to social innovation performance.

Social enterprise hybrids are embedded in wider networks of stakeholders when compared to commercial organizations (Low, 2006). For example, in addition to employees, suppliers, customers, and government agencies, social enterprise hybrids also build relationships with beneficiaries, volunteers, donors, philanthropists, and the wider community in which they operate (Lyon, 2012). Such diversity of stakeholder groups is reflected in goal plurality, and the adoption of trading to provide funds and resources to meet commercial, social, and environmental goals (Battilana et al., 2015; Dacin et al., 2011; Mair & Martì, 2006). While collaboration with stakeholders is fundamental to social enterprise hybrids, the extent to which stakeholder relationships and collaborations are related to social innovation performance is less clear (Phillips et al., 2015; van Wijk et al., 2018).

The relationships with external stakeholders provide conduits for the flow of external knowledge and ideas into the organization (Hostick-Boakye & Hothi, 2011; von Hippel, 1976, 1988). For example, external knowledge is sought by social enterprise hybrids to access resources, recruit employees and volunteers, and identify opportunities for trading and collaboration with intra-sector and cross-sector partners (Cooney, 2011; Le Ber & Branzei, 2010; Lyon, 2012). We would thus expect that the inflow of knowledge and ideas from relationships between a social enterprise hybrid and a wide range of stakeholders would raise awareness of market and government policy failures and, in turn, opportunities for social innovation (Lettice and Paraekh 2010). The moderation effect is such that the more social enterprise hybrids access external knowledge and ideas, the more they learn about opportunities for social innovation and the stronger the impact of commercial, social, and environmental goal orientation on social innovation performance. For example, stakeholder relationships were noted to be important for enhancing social innovations to assist the unemployed (Lyon, 2012), and to learn about how stakeholder energy needs could be served by implementing novel renewable energy technologies (Huybrechts & Haugh, 2017; Sengupta et al., 2020). The greater social enterprise hybrid openness to sources of knowledge and ideas, the greater the influence on goal orientation and social innovation performance.

H4a–c: Social enterprise hybrid openness to external sources of knowledge and ideas will positively moderate the relationship between commercial goal orientation (H4a), social goal orientation (H4b), environmental goal orientation (H4c), and social innovation performance.

## Methods and data

### Survey and data collection

To test our hypotheses, we developed a new survey to investigate social innovation and collected data from 380 social enterprise hybrids in the UK. The sample was selected from the population (Villeneuve-Smith & Temple, 2015) of 11,000 community interest companies (CICs). The CIC organizational form was established in 2005 to provide a company format that would enable, for the first time, the simultaneous pursuit of commercial activity and social and environmental mission.^2^ The CIC was initially created specifically for social enterprise hybrids that emerged during the period of government interest in the marketization of the social sector and the bottom-up revitalization of local communities (Low, 2006). The sample was derived from the register compiled by the CIC Registrar which lists every active CIC that has satisfied the requirements stipulated for this organizational form, i.e. incorporation and commitment to a specified community of interest. The sample was drawn from CICs across all regions of the UK, industrial sectors, and age of organization (2005–2015). Survey development, research process, and quantitative analysis used to test our hypotheses are discussed in detail in the following section.

To improve the reliability of the survey, we operationalized the measures using variables that had been successfully applied in previous social entrepreneurship and innovation empirical research. Validated questions were employed to measure commercial, social (Rao & Holt, 2005; Weber et al., 2015), environmental performance (Melnyk et al., 2003), and innovation (BIS, 2015). Where no comparable studies were found, the research team created variables and verified them in pre-testing and piloting.

The survey was pre-tested in two phases prior to data collection. First, in September 2014, the survey was submitted to a panel of 3 social entrepreneurs and the questions were then adjusted for clarity. Second, in October 2014, the survey was pilot tested online with a sample of 12 CICs to ensure that the questions were understood by the respondents, to check the feasibility and content validity of the survey and that empirical data would satisfy the research objectives. The results from the pilot survey are not included in the data analysis.

A statistically random sample was created in which a key respondent (Kumar et al., 1993), either the founder or managing director, was invited to complete the survey. The survey included questions to verify that the respondent was the key decision maker. We received a list of 9275 CICs from the CIC Regulator. One in four of the CICs, plus 200 CICs, were randomly selected. A total of 1259 invitations to complete the survey were sent to CICs in November 2014 and followed up with three reminders over a 1-month period. A further 1260 CICs were sent invitations in March 2015, again with three reminders following. Ninety-nine CICs had outdated contact information and could not be contacted. Four hundred thirty-one CICs completed the questionnaire but 51 questionnaires were unusable, leading to an initial sample of 380. Due to various missing values, the number of observations used in the actual econometric analysis was reduced to 288. A 15.7% response rate was achieved which compares well with other social enterprise surveys of 19% (Weber et al., 2015). Parametric and non-parametric tests found no evidence of response bias with regard to geographical location, industrial activity, and age of the social enterprises between respondents and non-respondents. Repeating the parametric and non-parametric tests between the 380 in the initial sample of replies and the 288 observations included in our models also found no evidence of response bias relating to geographical location, industrial activity, and age of the social enterprises.

Summary statistics are presented in Table 1. In Table 3 in the Appendix, we present a list of questions that were used in the construction of the variables. The demographic composition of the respondents was 56% female. Respondents were more likely to be the founder (68%) and/or the director (39%) of the CIC. Social enterprise hybrids with zero full-time employees were coded as 0.1 prior to the transformation to avoid the problem of lost observations (full-time). The log of zero is an undefined value so coding the zero values to 0.1 ensured that defined values could be obtained for all values when the log transformation was applied. Fifty-three percent of the social enterprises had zero full-time employees. Nineteen percent of the social enterprises had 1 full-time employee. The most full-time employees employed by a social enterprise was 60. Forty-four percent of the social enterprises had zero part-time employees. Ninety percent of the social enterprises had up to 6 part-time employees. Many social enterprises benefit from the work of volunteers and approximately 78% of the social enterprises had one or more volunteers. Sixty percent of the social enterprises had up to 5 volunteers. CIC’s main areas of activity according to the Standard Industrial Classification (SIC) 2007 code were most likely to be arts, entertainment and recreation (25%), education (20%), other service activities (18%), and human health and social work (15%). Accounting and financial services (4%), information and communication (4%), agriculture, forestry and fishing (4%), professional, scientific and technical (3%), real estate (2%), and advertising and support services (2%) were less mentioned activities.^3^

**Table 1:** Summary information and correlation matrix (*N* = 288)

	Mean	S.D	VIF	1	2	3	4	5	6	7	8	9	10	11	12	13	14	15	16	17	18	19
1. Innovation	2.26	1.90		1.00																		
2. Full-time	− 0.89	1.61	1.89	0.17^a^	1.00																	
3. Part-time	− 0.42	1.81	1.62	0.06	0.27^a^	1.00																
4. Volunteers	0.93	1.99	2.03	0.14^b^	− 0.10^c^	0.00	1.00															
5. Members	− 1.73	1.90	1.55	− 0.05	− 0.06	− 0.05	0.06	1.00														
6. CIC age	7.34	2.28	1.19	0.15^a^	− 0.12^b^	− 0.07	− 0.12^b^	− 0.05	1.00													
7. Founder	0.72	0.45	1.68	0.17^a^	− 0.01	0.06	0.01	− 0.13^b^	0.15^a^	1.00												
8. Gender	0.47	0.49	1.36	0.11^c^	0.04	− 0.03	− 0.06	0.03	− 0.06	− 0.03	1.00											
9. Young	0.21	0.41	1.49	0.17^a^	0.02	0.05	0.06	0.01	0.24^a^	0.08	− 0.03	1.00										
10. Middle	0.57	0.49	1.52	0.19^a^	− 0.01	0.07	− 0.11^c^	− 0.06	0.02	0.05	− 0.04	− 0.39^a^	1.00									
11. Older	0.22	0.41	1.50	0.17^a^	− 0.02	− 0.14^b^	0.06	0.07	− 0.27^a^	− 0.15^a^	0.08	− 0.28^a^	− 0.41^a^	1.00								
12. Compulsory	0.06	0.23	1.77	0.04	0.05	− 0.07	0.02	0.12^b^	0.05	0.08	0.05	− 0.05	− 0.01	0.06	1.00							
13. Advanced	0.12	0.32	1.73	0.15^a^	0.03	0.03	− 0.06	0.01	0.01	− 0.02	0.06	− 0.05	0.05	− 0.01	− 0.09	1.00						
14. Degree	0.36	0.48	1.89	0.17^a^	− 0.09	− 0.07	0.01	− 0.04	− 0.03	0.01	0.02	− 0.02	0.05	0.08	− 0.18^a^	− 0.27^a^	1.00					
15. Postgrad	0.45	0.49	1.94	0.19^a^	0.04	0.10^c^	0.03	0.04	− 0.02	0.11^c^	− 0.06	0.08	0.00	0.08	− 0.22^a^	− 0.32^a^	− 0.37^a^	1.00				
16. Financial	3.36	1.14	1.87	0.19^a^	0.23^a^	− 0.12^b^	0.02	0.08	− 0.12^b^	0.12^b^	− 0.09	− 0.14^b^	0.16^a^	0.12^b^	0.14^b^	0.03	0.07	− 0.06	1.00			
17. Social	4.68	0.53	1.68	0.18^a^	0.11^c^	0.06	− 0.09	− 0.12^b^	0.03	0.03	− 0.13^b^	0.16^a^	− 0.11^c^	0.15^a^	0.16^a^	− 0.14^b^	− 0.04	0.12^b^	0.03	1.00		
18. Environ	3.36	1.16	1.84	0.11^c^	0.08	− 0.04	0.18^a^	0.03	− 0.06	0.04	0.01	0.02	− 0.14^b^	0.14^b^	0.12^b^	0.06	− 0.05	− 0.02	0.12^b^	0.11^c^	1.00	
19. Openness	29.82	9.48	1.75	0.22^a^	0.15^a^	0.15^a^	0.14^b^	− 0.09	0.10	0.05	− 0.05	0.16^a^	0.19^a^	− 0.15^a^	− 0.12^b^	0.15^a^	0.17^a^	0.24^a^	0.18^a^	0.16^a^	0.15^a^	1.00

^c^*p* < 0.10; ^b^*p* < 0.05; ^a^*p* < 0.01

### Measures

#### Dependent variable

We looked to the innovation literature for guidance in question design (BIS, 2015). Measures employed to quantify innovation performance in commercial firms include formal indicators, such as patent registration, trademark and copyright protection, and informal indicators (Dahlander & Gann, 2010; West et al., 2014). Dziallas and Blind (2019) reviewed the range of innovation indicators throughout the innovation process. Bareghed et al. (2009) reviewed the difficulties in providing a multidisciplinary definition of innovation. Reliable formal measures are not (yet) available for social innovation. The respondents were asked if they had introduced new products or new services. Further, social innovation seeks its impact on social transformation, namely the impact of the social innovation on the social problem (Westley & Antadze, 2010). We therefore asked respondents to assess social innovation performance in terms of a measure that went from ‘0’ no new products or services; and for those respondents who had introduced a new product or service, the degree of impact of their social innovation activities using a five-point scale of ‘1’ (very low) to ‘5’ (very high) (Innovation).

#### Independent variables

To be categorized as a social enterprise hybrid, an organization must be more than financially sustainable; they need also to aim to create at least some social and environmental impact. Pursuing multiple goals thus defines their mission and is separate from measures of impact. Rawhouser et al. (2019) recently reviewed the variety of different definitions and approaches that have been used to measure social impact. Prior studies, such as the Global Entrepreneurship Monitor (http://www.gemconsortium.org), take on trust that self-defining as a social enterprise hybrid is sufficient guarantee of multiple goal orientation, or use an attention allocation scale originally devised for private-sector corporations (Stevens et al., 2015). In our study, we validated our sample by asking respondents to indicate the level of their *commercial*, *social*, and *environmental* goal orientation using a five-point scale from ‘1’ (not important) to ‘5’ (crucially important). Our measure thus captures practice rather than cognition.

*Openness* has been measured in different ways concerning the use of sources of external knowledge and ideas (Dahlander & Gann, 2010; Kostopoulos et al., 2011; Laursen & Salter, 2006). Since the extent of openness is generally assumed to be a continuous variable (Dahlander & Gann, 2010; Drechsler & Natter, 2012) we distinguish between the relative importance of sources of external knowledge and ideas on a scale of 1–5.

External sources of knowledge and ideas are defined as originating from organizations and individuals that are not employed by the responding firm (Dahlander & Gann, 2010). There is a large body of work which draws upon questions relating to the use and importance of a wide variety of sources of knowledge and ideas and firm innovation (e.g. Leiponen & Helfat, 2010; Love et al., 2014; Vivas & Barge-Gil, 2015). The specific external sources of knowledge and ideas were derived from previous literature and include informal networks, friends and relatives (Tödtling et al., 2009); customers, clients, and suppliers (Leiponen, 2000; von Hippel, 1988); users (Von Hippel, 1976; Voorberg et al., 2015); universities, colleges, and other educational institutions (Mina et al., 2014; Rodriguez et al., 2016); and consultants and providers of professional and financial services (Rodriguez et al., 2016). To these, we added sources for social innovation, namely beneficiaries, other third-sector organizations, social enterprises, and charities; and UK business information services, e.g. local, national, the CIC Regulator, and Her Majesty’s Revenue and Customs (HMRC). In addition, respondents were invited to specify and evaluate any sources of information not listed. The number of external sources of information where the respondents indicated that the impact was crucially important was calculated and gives a breadth-depth measure. We then multiplied openness by commercial goal orientation to create the first variable to test the moderating role of openness. This procedure was repeated for social goal orientation and then environmental goal orientation.

#### Control variables

The selection of control variables was guided by previous social entrepreneurship, innovation, and social innovation research. Innovation is associated with organization size (Mina et al., 2014), and specifically larger firms (Chesbrough, 2003). Our survey employs a logarithmic transformation of the total number of *full-time* employees. As social enterprise hybrids typically also supplement the full-time workforce with *part-time* employees and volunteers (Doherty et al., 2014; Farny et al., 2018), we adopted the same procedure to measure for part-time employees and *volunteer*s. We also requested information on *members* who pay a regular membership fee. Older social enterprise hybrids have had more time to build relationships with sources of external knowledge and ideas and we calculated *CIC age* from the year of incorporation to the date of the survey.

Demographic information was also gathered from the respondents. Organizational founders, in our case social entrepreneurs, have been imbued with heroic qualities concerning social innovation and change (Cajaiba-Santana, 2014; Hibbert et al., 2002). We distinguish between *founder* (code ‘1’) and otherwise (code ‘0’). For *gender*, the male respondents were coded as ‘1’ and the female respondents were coded as ‘0’. The age of the respondents was used to create three dummy variables as follows. Respondents aged 21–39 years old were coded as ‘1’ and otherwise ‘0’ (*young*)^4^; 40–60 years old were coded as ‘1’ and otherwise ‘0’ (*middle*); and 61 years and older were coded as ‘1’ and otherwise ‘0’ (*older*).

Finally, innovating firms need to have the competencies to take on board the value of new information from external sources and simultaneously assimilate and apply the new knowledge and ideas to develop new products, services, and processes (Dahlander & Gann, 2010; Drechsler & Natter, 2012; Kostopoulos et al., 2011). Since openness has been linked to employee human capital (Harison & Koski, 2010), respondents were asked to indicate their highest level of education. Respondents who left school aged 16 with a *compulsory* level of education were coded ‘1’ and otherwise ‘0’); respondents with *‘A’ levels* were coded as ‘1’ and otherwise ‘0’; respondents with a *degree* were coded ‘1’ and otherwise ‘0’; and respondents with *postgraduate* university qualifications were coded ‘1’ and otherwise ‘0’. The comparison group in the models is postgraduate university qualifications.

#### Common methods variance bias tests

In order to test for common methods variance bias (CMB), we used Harmon’s one-factor test (Podsakoff & Organ, 1986) and also the marker variable technique (Jarvenpaa & Majchrzak, 2008; Pavlou et al., 2007). Harmon’s one-factor test has been applied by Love et al. (2014) and thus there is precedent for its use. We included all independent and control variables in Harmon’s one-factor test. If the un-rotated factor accounts for a substantial proportion, 50% plus, of the total variance, then CMB is a problem (Love et al., 2014). We found that the first un-rotated factor accounted for approximately one-fifth of the total variance and thus interpreted this result as evidence that CMB is not a problem in our models.

Harmon’s test has, however, been argued to be insufficient to test for potential CMB (Podsakoff et al., 2003), and so, we also employed a marker test (Lindell & Whitney, 2001). In essence, there are two possible procedures with a marker test. First, the researchers need to identify a variable which is not theoretically related to at least one variable in a study (Jarvenpaa & Majchrzak, 2008; Pavlou et al., 2007). Second, if the former option is not possible, then researchers need to use the variable with the lowest correlation with other variables to become the marker variable. We followed the second route and found that there is no evidence of CMB (Lindell & Whitney, 2001). Podsakoff et al. (2003) have provided an extensive review of techniques available to control for the effects of CMB, and we acknowledge that the marker test is not without problems (see Richardson et al., 2009). Notwithstanding these points, we have applied several tests to validate our use of CMB, and as indicated below, the correlation matrix also provides further evidence that the results reported in the next section are appropriate.

### Model specification

The dependent variable Innovation is a categorical variable and this suggests that an ordered logit or probit regression technique should be utilized (Greene, 2012; Long, 1997). Below we report the ordered logit regression models which have been estimated using STATA (StataCorp, 2013).

## Results

Examination of the correlation matrix (see Table 1) showed no evidence of multicollinearity. Table 2 presents the results of the ordered logit models which test the relationships between commercial, social, and environmental goal orientation and social innovation performance, and the moderating role of openness to external sources of knowledge and ideas. Our model specifications are based upon controlling for variables which have been found to be important in the literature on innovation and openness. Model 1 includes the control variables. In model 2, we augment model 1 with commercial goal orientation. In model 3, social goal orientation is added to the control variables. In model 4, the control variables are augmented with environmental goal orientation. In model 5, the openness variable and the openness variable times commercial goal orientation is added. The openness variable and the openness variable times social goal orientation is added in model 6. Lastly, the openness variable and the openness variable times environmental goal orientation is added in model 7. The likelihood ratio chi-squared test is statistically significant at the 0.01 level in all 7 models (Greene, 2012).

**Table 2:** Ordered Logit models of the social innovation of the social enterprise hybrids (*N* = 288)

	Model 1		Model 2		Model 3		Model 4		Model 5		Model 6		Model 7	
	Coef	S. E	Coef	S. E	Coef	S. E	Coef	S. E	Coef	S. E	Coef	S. E	Coef	S. E
Full-time	**0.18**^**b**^	**0.07**	**0.16**^**b**^	**0.07**	**0.19**^**b**^	**0.07**	**0.18**^**b**^	**0.07**	**0.18**^**b**^	**0.07**	**0.17**^**b**^	**0.07**	**0.18**^**b**^	**0.08**
Part-time	0.06	0.08	0.04	0.06	0.06	0.06	0.05	0.07	0.05	0.07	0.06	0.07	0.08	0.09
Volunteers	**0.12**^**b**^	**0.06**	**0.12**^**b**^	**0.06**	**0.12**^**b**^	**0.06**	**0.12**^**b**^	**0.06**	**0.12**^**b**^	**0.06**	**0.12**^**b**^	**0.06**	**0.12**^**b**^	**0.06**
Members	− 0.01	0.05	− 0.01	0.05	− 0.01	0.05	− 0.01	0.05	− 0.01	0.05	− 0.02	0.06	− 0.01	0.06
CIC age	**0.05**^**b**^	**0.02**	**0.07**^**b**^	**0.03**	**0.05**^**b**^	**0.02**	**0.05**^**b**^	**0.02**	**0.05**^**b**^	**0.02**	**0.07**^**b**^	**0.03**	**0.06**^**b**^	**0.03**
Founder	**0.37**^**b**^	**0.17**	**0.34**^**b**^	**0.17**	**0.37**^**b**^	**0.18**	**0.36**^**b**^	**0.18**	**0.36**^**b**^	**0.18**	**0.35**^**b**^	**0.18**	**0.34**^**b**^	**0.17**
Gender	**0.36**^**c**^	**0.21**	**0.38**^**c**^	**0.21**	0.35	0.21	**0.36**^**c**^	**0.21**	**0.36**^**c**^	**0.21**	0.34	0.22	**0.36**^**c**^	**0.22**
Middle	0.21	0.28	0.26	0.28	0.23	0.29	0.20	0.28	0.20	0.28	0.23	0.29	0.22	0.29
Older	0.19	0.35	0.23	0.35	0.20	0.35	0.21	0.35	0.20	0.35	0.22	0.35	0.21	0.35
Compulsory	− 0.05	0.46	− 0.07	0.46	− 0.05	0.46	− 0.08	0.46	− 0.08	0.46	− 0.08	0.46	− 0.06	0.47
Advanced	** − 1.12**^**a**^	**0.35**	** − 1.16**^**a**^	**0.34**	** − 1.13**^**a**^	**0.35**	** − 1.15**^**a**^	**0.35**	** − 1.15**^**a**^	**0.35**	** − 1.15**^**a**^	**0.36**	** − 1.19**^**a**^	**0.36**
Degree	** − 0.54**^**b**^	**0.24**	** − 0.58**^**b**^	**0.24**	** − 0.54**^**b**^	**0.24**	** − 0.53**^**b**^	**0.24**	** − 0.53**^**b**^	**0.24**	** − 0.48**^**b**^	**0.24**	** − 0.48**^**b**^	**0.24**
Commercial	–	–	**0.15**^**b**^	**0.07**	–	–	–	–	**0.16**^**b**^	**0.07**	–	–	–	–
Social	–	–	–	–	**0.19**^**b**^	**0.09**	–	–	–	–	**0.20**^**b**^	**0.09**	–	–
Environ	–	–	–	–	–	–	** − **0.10	0.10	–	–	–	–	-0.09	0.20
Openness	–	–	–	–	–	–	–	–	**0.18**^**b**^	**0.08**	**0.17**^**b**^	**0.08**	**0.20**^**b**^	**0.09**
Commercial*Openness	–	–	–	–	–	–	–	–	**0.15**^**a**^	**0.04**	–	–	–	–
Social*Openness	–	–	–	–	–	–	–	–	–	–	**0.12**^**b**^	**0.06**	–	–
Environ*Openness	–	–	–	–	–	–	–	–	–	–	–	–	-0.03	0.03
Log likelihood	− 478.82		− 477.70		− 477.79		− 478.33		− 474.33		− 470.12		− 472.03	
Likelihood ratio	**31.65**^**a**^		**33.90**^**a**^		**32.72**^**a**^		**32.63**^**a**^		**34.63**^**a**^		**36.84**^**a**^		**35.23**^**a**^	
McFadden’s *R*^2^	0.1511		0.1614		0.1683		0.1632		0.2199		0.2322		0.2249	

Postgraduate is the comparison level of education. Young is the comparison age of the key decision maker. ^c^*p* < 0.10; ^b^*p* < 0.05; ^a^*p* < 0.01

There are many ways to calculate the goodness of fit, an *R*^2^ measure in logistic regression and amongst ordered logit applications. Mittlbock and Schemper (1996) reviewed 12 different measures and Menard (2000) assessed further measures. All the measures of goodness of fit have their weaknesses (Allison, 2013; Greene, 2012; Liu, 2015; Long & Freese, 2014) but the most widely used measures are the McFadden (1974) and Cox and Snell (1989) goodness of fit. Following precedent, we have reported the McFadden measure of goodness of fit. Given the way that the McFadden (1974) measure is calculated, the values in an ordered logit context are typically much lower than an *R*^2^ measure in an OLS application. Louviere et al. (2000) indicate that a McFadden (1974) measure of between 0.2 and 0.4 is considered a very good fit of the model and that the aforementioned values have equivalence of 0.7 to 0.9 in a linear context. The McFadden’s *R*^2^ is 0.1511 in Model 1 (StataCorp, 2013). The McFadden’s *R*^2^ is 0.2199 in model 5, 0.2322 in model 6, and 0.2249 in model 7.

In models 2 and 5, commercial goal orientation is statistically significant at the 0.05 level. Commercial goal orientation of social enterprise hybrids is positively related to social innovation performance. The empirical data thus supports hypothesis H1. In models 3 and 6, social goal orientation is positively statistically significant at the 0.05 level. Thus, the level of social goal orientation of social enterprise hybrids is systematically statistically related to social innovation performance. The empirical data provides support for hypothesis H2. In models 4 and 7, environmental goal orientation is negatively related to social innovation, but it is not statistically significant at the 0.10 level, or better. Thus, there is no systematic relationship between environmental goal orientation and social innovation performance; therefore, hypothesis H3 is not supported by the empirical data.

In model 5, we see that the interaction of the openness and commercial goal orientation variables is statistically significant at the 0.05 level. Thus, there is evidence to support hypothesis H4a on the moderating role of openness upon commercial goal orientation and social innovation performance. In model 6, the interaction of openness and social goal orientation is also statistically significant at the 0.05 level. The result indicates evidence to support hypothesis H4b on the moderating role of openness upon social goal orientation and social innovation performance. However, in model 7, the interaction of the openness and environmental goal orientation variables appears with a negative signed coefficient and is not statistically significant at the 0.10 level or better. Thus, there is no evidence to support hypothesis H4c.

In relation to the control variables, we find that several of the variables are consistently statistically significant in the models. First, as the number of full-time employees and also the number of volunteers in the social enterprise hybrids increases, this is positively associated with social innovation performance. Second, social innovation performance is also strongly associated with age of the social enterprise hybrids as older social enterprise hybrids are positively associated with social innovation performance. Thirdly, having the founder as the key decision maker in social enterprise hybrids is associated with social innovation performance. Fourthly, the education of the key decision makers is systematically related to social innovation performance. Key decision makers with an advanced level of education, or a degree, have a negative association with social innovation performance compared to those social enterprise hybrids where the key decision maker has a postgraduate level of education.

As a sensitivity analysis, we re-ran models where the dependent variable (Innovation) and the independent variable Openness were recalculated as dummy variables where scores of 5 or 5 and 4 were coded as 1 and otherwise 0. These alternative binary logit models generated qualitatively similar results, with the exception of the model where scores of 4 and 5 were combined and the moderating effects for H4a and b were still positive but no longer significant. We also tested to see if there are curvilinear relationships between our independent variables against social innovation performance by including quadratic terms, but these are not statistically significant. Thus, we found no evidence of curvilinear relationships.

## Discussion

The social enterprise hybrid mission is to pursue commercial, social, and environmental goals and, when successful, they contribute to creating economies that are financially, socially, and environmentally sustainable. Our investigation portrays a complex set of relationships between social enterprise hybrid goal orientation and social innovation performance. The generation of income from trading distinguishes social enterprise hybrids from other social purpose organizations that are grant dependent, such as NGOs and charities. Grant dependence restricts the organizational use of funds to those specified in the terms and conditions of the donation. For social enterprise hybrids, commercial strategies are fundamental to generate the flow of funds into the organization and confer on them the freedom to decide where and how to invest unrestricted income. Successful commercial strategies would thus lead to funds being available to invest in social innovation performance. For social enterprise hybrids, social goal orientation is also integral to securing legitimacy (Dart, 2004) and the close relationships between social enterprise hybrids and users enable them to generate new products and services that are tailored to market demand. In some organizations, legitimacy is further strengthened by user involvement in cocreating new products and services (Voorberg et al., 2015). Thus, our findings support the positive relationship between mission and social innovation performance (McDonald, 2007), but only for commercial and social goal orientation.

To illustrate, social enterprise hybrids such as Musica Music and Wellbeing CIC support people living with dementia and their carers by providing opportunities to engage with music in domestic and care homes, day centres, and hospitals (NatWest SE100, 2020). Trading income is generated from fees for workshops and enrolment in training courses. The inflow of knowledge and ideas is secured from relationships with public sector organizations, partners, clients, and service users. External knowledge and ideas are employed to innovate social innovations such as online coaching, online service delivery, and free online workshops for music in dementia care.

Commercial success is integral to the pursuit of social and environmental goals (Moizer & Tracey, 2010) irrespective of whether business models to achieve commercial and other goals are either fully integrated or dependent on cross-subsidization between different business units. Although commercial mission seeks to identify and exploit market opportunities and social mission is oriented to identifying and servicing unmet social needs, both strategies rely on deep knowledge of the requirements of external stakeholders. Successful commercial strategies are dependent on close relationships with suppliers, distributors, and customers to better understand which products and services are needed by customers, how much they are willing to pay, where purchases will be made, etc. (Chesbrough, 2003, 2006a; Von Hippel, 1988; West et al., 2014). Successful strategies for achieving social goals are dependent upon deep understanding of market and government policy failures, availability of resources, and client needs. Information about social needs is acquired from the flow of knowledge and ideas that is mediated between the social enterprise hybrid and external stakeholders. Thus, close relationships with external sources of knowledge and ideas are integral to developing strategies for commercial and social goal orientation. As such, our results complement recent case studies of social innovation (Chesbrough & Minin, 2014) by finding a positive and significant relationship between openness and social innovation performance.

For example, Assisted Homes CIC (London) provides accommodation and support services to the homeless and pending homeless, i.e. rough sleepers, sofa-surfers, and people fleeing domestic abuse (CIC Regulator Report, 2017/18). In addition to finding clients a safe and secure place to stay, Assisted Homes CIC leverages its network connections to provide tailored medical, social, and life skill support to help clients build a more stable future. Information about how best to provide support services is gathered from relationships with consultants knowledgeable about the availability of specialist services, and directly from users. This information is employed to innovate a bespoke support plan for clients.

Our result concerning the lack of a relationship between environmental goal orientation and social innovation performance is surprising but may be attributable to the nature of social innovations that are designed to address environmental problems. The resource constraints faced by social enterprise hybrids suggest that they are more likely to adopt innovations to address environmental issues, such as green technologies and organic and nature conservation standards, which have been developed by other organizations. Examples of such innovations include bio-digestion, wind energy, photovoltaics, and biomass and biofuels (Sengupta et al., 2020; Surie, 2017) and environmental management systems (Batle et al., 2018). Where extant social innovations designed to solve environmental problems are working effectively, we suggest that strategies of learning and replication (Luke & Chu, 2013) may be resource-efficient ways for social enterprise hybrids to achieve environmental goals as the imitative adoption of extant social innovations might incur less risk than investment of their own funds (Chalmers, 2012). To illustrate, Scott-Cato and Hillier (2010) consider the spread of the Transition Town model as an example of how climate-related social innovations have diffused by adoption (Vickers & Lyon, 2014). Social innovation to address environmental problems by imitation is a lower cost strategy than investing in developing innovations to respond to environmental problems afresh.

For example, the aim of Yorkshire Energy Service CIC, trading as Yes Energy Solutions Ltd., is to reduce CO_2_ emissions and alleviate fuel poverty. Income is generated from contracts with energy companies obliged to comply with energy efficiency targets (CIC Regulator Report, 2018/19). In partnership with a wide range of public sector organizations, e.g. the Energy Companies Obligation (ECO) Scheme; social enterprises, e.g. Housing Associations; and NGOs, e.g. the Fuel Poor Energy Network Scheme, consumers are taught a repertoire of existing standard techniques to increase their fuel efficiency. Social innovation in services and service delivery is implemented to respond to new climate and energy policies, and regulatory changes.

Further, social innovation for environmental transformation might also be more technology-based than those for social and welfare services and hence can be appropriated more readily than social innovations that require tailoring for individual and community use (Batle et al., 2018; Bhatt et al., 2016). With the exception of accidents and catastrophes, organizations are more able to exercise control over their environmental goal orientation than social goal orientation through the design, implementation, and monitoring of environmentally-sensitive products, services, and processes. Thus, we propose that environmental goal orientation is also more dependent on the monitoring and control of internal processes and procedures than on building close relationships with external stakeholders.

Eco Larder CIC is a zero-waste supermarket in Edinburgh established to reduce single-use packaging in food retailing and raise customer awareness of ways to live a zero-waste lifestyle (CIC Regulator Report, 2018/19). Income is generated in four ways, first, the sale of products in zero-waste supermarkets; second, fees from workshops that teach customers how to make their own household and bathroom essentials; third, fees from business consulting services; and finally, the sale of products made from material collected from beach clean-ups. During the COVID 19 pandemic, their control of internal processes and procedures helped them to achieve social innovations that included a free home delivery service that used electric cargo bikes, and a new partnership with a food bank to provide meals to the disadvantaged.

Our results also frame the purposive inflow of knowledge and ideas to be beneficial to social innovation performance. In commercial firms, innovativeness is positively related to profitability (Leiponen, 2000) and returns to investors (Sood & Tellis, 2009). The governance structure of social enterprise hybrids, however, directs returns to beneficiaries, communities, and social transformation, and thus, performance is evaluated in relation to the positive effect of novel solutions on social problems. The impact of external sources of knowledge and ideas on environmental strategies may be in response to new regulations, and the impact less direct than found in strategies for commercial and social goal orientation. For example, when commercial and environmental strategies are integrated, e.g. upcycling of waste into new products, recycling used products for re-sale or gifting to new customers, then environmental goal orientation might be assisted by the external sources of information advice employed to facilitate commercial goal orientation.

North Wales Recycle IT CIC provides the only secure and professional recycling, reuse, and disposal of IT equipment for individuals, families, and organizations in North Wales. Income is generated from the collection of IT equipment, and refurbished IT equipment is provided to charities, community groups, low-income families, the long-term unemployed, and new businesses. Stakeholder relations with suppliers, end users, the local community, and local government provide knowledge and ideas. For example, the CIC enables organizations to comply with the Waste Electrical and Electronic Equipment (WEEE) Regulations by managing secure data destruction from IT hardware, and the safe disposal of IT equipment. Recycling reduces the amount of IT equipment sent to landfills.

Although competition between commercial firms increases efficiency and effectiveness, the cultural values of social enterprise hybrids prioritize collaboration to maximise social impact above competition to maximise profits (Le Ber & Branzei, 2010; Santos et al., 2015). Recent research has noted how social enterprise hybrids can serve as incubators for new practices that can then be scaled up through cross-sector collaborations (Lee & Jay, 2015). In this way, the collaborative values of social enterprise hybrids may cross institutional boundaries and infuse commercial firms (Lee & Jay, 2015).

Finally, our research also contributes to widening the understanding of the scope of activities that comprise hybrid organizing (Battilana & Lee, 2014). Hybrid organizing describes the “activities, structures, processes and meanings” (Battilana & Lee, 2014, p. 397) of organizational forms that bridge different institutional domains (Tracey et al., 2011). A review of the literature concluded that the dimensions of hybrid organizing consisted of inter-organizational relationships, culture, organizational design, workforce composition, and organizational activities (Battilana & Lee, 2014). In their description of hybrid, organizing inter-organizational relationships is related to accessing financial resources and markets (pp. 420–21). The empirical results from our study, however, find that openness to knowledge and ideas from external stakeholders is significantly related to economic and social goal orientation and social innovation performance. From our research, we propose that the activities which comprise hybrid organizing be extended to include openness to external stakeholders to secure a wider set of benefits beyond access to finance and markets.

## Conclusion

The intractability of poverty, inequality, and the impact of climate change has increased attention on the potential of social innovation performance to generate long-term solutions to social and environmental challenges. Such grand challenges have attracted the attention of a wide range of different organizational forms as they adapt to the institutional and societal expectations and environmental conditions of the twenty-first century.^5^ Insights into the influence of goal orientation on social innovation and the impact of openness on these relationships have been limited to date and our study is the first to shed light on the social enterprise hybrid and social innovation performance. The results have found that social enterprise hybrids’ commercial and social goal orientation are positively related to social innovation performance. In relation to openness, external sources of knowledge and ideas positively moderate commercial and social goal orientation and social innovation performance. The results contribute to our understanding of social innovation, openness, and hybrid organizing.

The social entrepreneurship literature suggests that the mission of many social enterprise hybrids is distinctly social and therefore the social enterprise hybrid is sustainable only to the extent that financial revenue can achieve social goals (Dacin et al., 2011). In common with commercial firms, the use of a larger pool of external knowledge and ideas is beneficial to the commercial performance of social enterprise hybrids. The strong moderating effect of openness on commercial and social goal orientation suggests that social enterprise hybrids benefit from drawing upon external knowledge and ideas as this favourably positions them for developing social innovation performances.

Many benefits have been advocated for innovation (Dahlander & Gann, 2010), however, agreement on the conditions whereby firms that invest in acquiring external sources of information outperform others has yet to be secured. In our study, openness and social innovation performance are positively related, and thus, the innovation imperative of commercial firms is also shared by social enterprise hybrids.

Implications of our research for practice and also for understanding the practical implications of a social enterprise hybrid business model include encouraging social enterprise hybrids to appreciate the strategic benefits of investing resources in building relationships with external stakeholders. Relationships with external stakeholders provide conduits for information about entrepreneurial opportunities arising from market and government policy failures, ideas for how such failures can be responded to, and how to secure competitive advantage from leveraging the benefits of collaborating with partners.

We conclude with three suggestions for further research that arise from the limitations of the research. First, the data in our study is cross-sectional and thus we are unable to isolate causality between goal orientation, openness, and social innovation. Furthermore, cross-sectional research does not readily permit detailed analysis of learning effects that may take several years for the benefits to become apparent (Love et al., 2014). Although our paper offers an important first step in relating goal orientation, openness, and social innovation performance in social enterprise hybrids, a longitudinal panel study would isolate causality between the variables. Second, the finding that openness is associated with commercial and social goal orientation, but not environmental goal orientation, is intriguing. We do not find that the strong body of research that advocates the benefits of openness applies to environmental goal orientation. Further research to unpack the complexities between social enterprise hybrids and environmental goal orientation would extend knowledge on variation in openness impact.^6^ This may require qualitative, case study research (Datta, 2011) to unpack the influences on environmental goal orientation. Finally, our study investigates the inflow of knowledge and ideas and employs an incident measure of social innovation performance. Further research that investigates social enterprise hybrids and the mechanisms and impact of knowledge spillover and formal, or third party, measures of social innovation performance would contribute to knowledge about how the collaborative values of the social economy foster, or impede, open social innovation.

## Appendix

**Table 3:** Questions used in the survey

Variable	Question
Innovation	“Please indicate the impact of the products or services innovation on the CIC.” Respondents were given a five-point scale from ‘1’ (not important) to ‘5’ (crucially important) Source: DBIS, 2015
Commercial	“Please indicate the importance of financial goals in this CIC.” Respondents were given a five-point scale from ‘1’ (not important) to ‘5’ (crucially important). Source: Adapted from Weber and Lambrich (2013)
Social	“Please indicate the importance of social goals in this CIC.” Respondents were given a five-point scale from ‘1’ (not important) to ‘5’ (crucially important). Source: Adapted from Weber and Lambrich (2013)
Environ	“Please indicate the importance of environmental goals in this CIC.” Respondents were given a five-point scale from ‘1’ (not important) to ‘5’ (crucially important). Source: Adapted from Weber and Lambrich (2013)
Openness	“Have the following as sources of information, advice and support been used with reference to this CIC? Please indicate the importance of each source that you have used.” Respondents were given a five-point scale from ‘1’ (not important) to ‘5’ (crucially important). Respondents were given a list of the following sources: friends and relatives; employees or volunteers; customers or clients; beneficiaries or users; business associates; University or College; consultants; suppliers; CICs, social enterprises or charities; professional services e.g. accountant, solicitor or lawyer; financial services providers e.g. bank, venture capitalists or business angels; a UK local authority e.g. a Council; UK national government sources e.g. BIS, CIC Regulator, HMRC; Other, please specify Source: Adapted from DBIS (2015) and Hunt et al. (2009)
Full-time	“Please indicate the current number of full-time people at the CIC. If zero, please indicate 0.” Adapted from Hunt et al. (2009)
Part-time	“Please indicate the current number of part-time people at the CIC. If zero, please indicate 0.” Adapted from Hunt et al. (2009)
Volunteers	“Please indicate the current number of volunteers at the CIC. If zero, please indicate 0.” Adapted from Hunt et al. (2009)
Members	“Please indicate the current number of members at the CIC. If zero, please indicate 0.” Adapted from Hunt et al. (2009)
CIC Age	“In which year was the CIC founded?” Adapted from Hunt et al. (2009)
Founder	“What is your position in this CIC? Please select all options which apply.” The respondents were given a series of options of: Founder, Managing Director, Director, Other please specify. Respondents who indicated that they were a founder were coded as ‘1’ and otherwise ‘0’ (*Founder*). Source: Adapted from Hunt et al. (2009)
Gender	“What is your gender? Male or Female?” The male respondents were coded as ‘1’ and the female respondents were coded as ‘0’ (*Gender*). Source: Adapted from Hunt et al. (2009)
Young, middle and older	“What is your age in years?” Respondents aged 21–39 years old were coded as ‘1’ and otherwise ‘0’ (*Young*); 40–60 years old were coded as ‘1’ and otherwise ‘0’ (*Middle*) and 61 years and older were coded as ‘1’ and otherwise ‘0’ (*Older*). Source: Adapted from Hunt et al. (2009)
Compulsory, advanced, degree, postgraduate	“What is your highest level of education? “Respondents were given a series of options of: No formal qualifications; GCSE/’O’ level or equivalent (*Compulsory*), ‘A’ level or equivalent (*Advanced*), Degree level (*Degree*); Postgraduate degree, postgraduate diploma or certificate, doctorate (*Postgraduate*); and Other please specify. Source: Adapted from Hunt et al. (2009)

## Footnotes

1 See Liket and Maas (2016) for a review of the measurement of philanthropic impacts in corporations.

2 The legislation to establish the CIC organizational form was introduced in the UK in the 2005 Companies (Audit, Investigations and Community Enterprise) Act. The government’s aim in creating the CIC was to establish a corporate form for social enterprise that could be set up quickly and easily and that would “combine the flexibility and familiarity of the company form” with a strong brand for social enterprise (DTI 2005, Sect. 7.1). CICs combine aspects of company law i.e. limited liability, the capacity to issue shares to raise capital and distribute dividends to shareholders (within limits specified by the Regulator of CICs) with characteristics of non-profit organizations i.e., social and environmental purpose. The community benefit requirement is that at the point of registration the CIC must generate benefits (directly or indirectly) to a client base wider than its membership (BIS 2015, p. 16*).* CIC organizations are thus akin to hybrid structures in that they bridge the private and the non-profit sectors. By the end of 2005, 83 CICs had been registered in England and this rose to about 11,000 in 2015.

3 Wholesale and retail trade; water supply, sewage, and waste management; manufacturing; financial and insurance; and construction together accounted for approximately 3% of social enterprises.

4 The youngest age of the respondents was 21 years old.

5 See Barney and Rangan (2022) for an interesting recent comment on these challenges in light of the role of, on the one hand, non-market institutions and, on the other hand, for-profit firms that could adopt both economic and social goals and generate innovations to achieve these goals.

6 As suggested by one of the reviewers, social enterprise hybrids might also be more socially oriented and less focused on the environmental component of social innovation.
